# The product of fasting plasma glucose and triglycerides improves risk prediction of type 2 diabetes in middle-aged Koreans

**DOI:** 10.1186/s12902-018-0259-x

**Published:** 2018-05-30

**Authors:** Joung-Won Lee, Nam-Kyoo Lim, Hyun-Young Park

**Affiliations:** 1 Division of Cardiovascular Diseases, Center for Biomedical Sciences, Korea National Institute of Health, 187 Osongsaengmyeng 2-ro, Osong-eup, Heungdeok-gu, Cheongju-si, Chungcheongbuk-do 361-951 South Korea; 2 Department of Public Health Sciences, Graduate School, Korea University, Seoul, South Korea

**Keywords:** TyG index, Type 2 diabetes mellitus, Risk model

## Abstract

**Background:**

Screening for risk of type 2 diabetes mellitus (T2DM) is an important public health issue. Previous studies report that fasting plasma glucose (FPG) and triglyceride (TG)-related indices, such as lipid accumulation product (LAP) and the product of fasting glucose and triglyceride (TyG index), are associated with incident T2DM. We aimed to evaluate whether FPG or TG-related indices can improve the predictive ability of a diabetes risk model for middle-aged Koreans.

**Methods:**

7708 Koreans aged 40–69 years without diabetes at baseline were eligible from the Korean Genome and Epidemiology Study. The overall cumulative incidence of T2DM was 21.1% (766 cases) in men and 19.6% (797 cases) in women. Therefore, the overall cumulative incidence of T2DM was 20.3% (1563 cases). Multiple logistic regression analysis was conducted to compare the odds ratios (ORs) for incident T2DM for each index. The area under the receiver operating characteristic curve (AROC), continuous net reclassification improvement (cNRI), and integrated discrimination improvement (IDI) were calculated when each measure was added to the basic risk model for diabetes.

**Results:**

All the TG-related indices and FPG were more strongly associated with incident T2DM than WC in our study population. The adjusted ORs for the highest quartiles of WC, TG, FPG, LAP, and TyG index compared to the lowest, were 1.64 (95% CI, 1.13–2.38), 2.03 (1.59–2.61), 3.85 (2.99–4.97), 2.47 (1.82–3.34), and 2.79 (2.16–3.60) in men, and 1.17 (0.83–1.65), 2.42 (1.90–3.08), 2.15 (1.71–2.71), 2.44 (1.82–3.26), and 2.85 (2.22–3.66) in women, respectively. The addition of TG-related parameters or FPG, but not WC, to the basic risk model for T2DM (including age, body mass index, family history of diabetes, hypertension, current smoking, current drinking, and regular exercise) significantly increased cNRI, IDI, and AROC in both sexes.

**Conclusions:**

Adding either TyG index or FPG into the basic risk model for T2DM increases its prediction and reclassification ability. Compared to FPG, TyG index was a more robust T2DM predictor in the stratified sex and fasting glucose level. Therefore, TyG index should be considered as a screening tool for identification of people at high risk for T2DM in practice.

## Background

Type 2 diabetes mellitus (T2DM) is one of the most prevalent non-communicable diseases in the middle-aged population worldwide, largely because of recent changes in diet and lifestyle [1, 2]. In the Korean National Health and Nutrition Examination Surveys (KNHANES), the prevalence of diabetes among adults aged 30 and over was found to have slightly increased, from 12.4% in 2011 to 13.0% in 2014 [3, 4]. According to an estimate by the International Diabetes Federation, 46.3% of cases of diabetes in Koreans aged 20–79 were undiagnosed in 2015 [5]. However, lifestyle intervention can reduce the risk of incident T2DM and mortality in individuals at high risk of diabetes [6, 7]. Therefore, it is important to screen high-risk individuals for T2DM regularly to ensure early diagnosis. For this reason, risk models for diabetes have been proposed in previous studies, and, recently, T2DM risk prediction models have been reported in Korea [8–14].

Obesity is the most significant risk factor for incident T2DM [15, 16]. Body mass index (BMI) has been used as a surrogate marker for obesity and included as one of the variables in most risk models for T2DM [9, 11–14]. However, BMI does not reflect central obesity. Lee et al. selected waist circumference (WC) instead of BMI in their diabetes risk model considering its association with diabetes [10]. In the systematic review, more than 30% of the diabetes risk models stating its components included both BMI and WC [8]. To improve the prediction ability of a risk model for incident T2DM, blood parameters are also frequently included [8].

Also, the increase of fasting plasma glucose (FPG) in the normal range is associated with increased incident T2DM [17]. Of these, serum triglyceride (TG) has been used to identify people at high risk for T2DM, alongside obesity [18]. In addition, lipid accumulation product (LAP) and the product of fasting plasma glucose and triglyceride (TyG index), composite indices including TG, have been proposed as predictors of T2DM [19, 20]. In particular, TyG index has been used as a marker of insulin resistance [19]. Although the simple diabetes risk model is convenient for self-assessment, more accurate prediction models that include blood parameters are also required to facilitate more accurate clinical consultations [8]. In Korea, most people are registered with the National Health Insurance (NHI), which provides biannual medical check-ups for middle-aged people, including the measurement of key blood parameters [21]. Therefore, risk models for incident T2DM that are based on the data obtained from these medical check-ups would be of great use for the prediction of risk of future T2DM. To date, few studies have been undertaken in Korea that compare the predictive ability for incident T2DM of the simple model and composite models, which include blood test results [11, 22]. Recently, a risk model for T2DM that included blood test results was proposed based on cohort data, and its reclassification ability was significantly improved when glycated hemoglobin (HbA_1c_) was included [11]. However, HbA_1c_ is not assessed in the routine health examination and this study did not consider reclassification ability when other blood test results apart from HbA_1c_ were included in the T2DM risk model [23].

Therefore, in the present study, we aimed to identify which of the TG-related indices that can be derived from general check-up data would improve the prediction ability of the simple T2DM risk model in middle-aged Koreans.

## Methods

### Study population

The Korean Genome and Epidemiology Study (KoGES) consists of a gene-environment model and population-based studies [24]. KoGES: Ansan and Ansung study is an ongoing prospective cohort study conducted in urban (Ansan) and rural (Ansung) areas in Korea with biennial follow-ups, which started in 2001. 10038 people underwent an initial examination, and 9001 subjects were included after exclusion of 1037 who refused to participate or died. Thirty-five participants were not suitable for the present study because of their age, and subjects with a history of diabetes at baseline or incomplete data were also excluded. Finally, 7708 people aged 40 to 69 years remained eligible for the current study. Written informed consent was obtained from all subjects. The Institutional Review Board of the Korean Centers for Disease Control and Prevention approved the study protocol.

### Measurements and surveys

Height and weight were measured to the nearest 0.1 cm and 0.1 kg using a digital stadiometer and a scale, respectively. Resting blood pressure while sitting was measured by trained technicians using a standard mercury sphygmomanometer. Blood samples were collected after fasting for at least 8 h. The Friedewald formula was used to indirectly estimate low-density lipoprotein cholesterol levels in subjects with plasma TG < 400 mg/dl [25]. Diabetes was defined by FPG > 126 mg/dl, 2 h post-challenge plasma glucose > 200 mg/dl, HbA_1c_ > 6.5%, or prescription for anti-diabetic medication [26]. Subjects were questioned by trained interviewers regarding their socio-demographics, family history of diabetes, and lifestyle factors including smoking and alcohol consumption. The subjects’ smoking and alcohol consumption status was subdivided according to their past and present habits. Regular exercise was defined as subjects’ exercise was over 90 min as the sum of moderate and vigorous physical activity a day [27].

LAP and TyG index were calculated as follows:

$$ {\displaystyle \begin{array}{c}\mathrm{LAP}\;\mathrm{for}\;\mathrm{men}=\left[\mathrm{WC}\left(\mathrm{cm}\right)\hbox{-} 65\right]\times \mathrm{TG}\left(\mathrm{mmol}/\mathrm{L}\right)\\ {}\mathrm{LAP}\;\mathrm{for}\kern0.17em \mathrm{women}=\left[\mathrm{WC}\left(\mathrm{cm}\right)\hbox{-} 58\right]\times \mathrm{TG}\left(\mathrm{mmol}/\mathrm{L}\right)\\ {}\mathrm{TyG}\;\mathrm{index}=\ln \left[\mathrm{TG}\left(\mathrm{mg}/\mathrm{dl}\right)\times \mathrm{FPG}\left(\mathrm{mg}/\mathrm{dl}\right)/2\right]\end{array}} $$

### Statistical analysis

Data are expressed as numbers and proportions for discrete variables and mean ± SD for continuous variables, and were analyzed using Chi-square and Student’s t-tests, respectively.

Based on the maximized Youden index, we calculated sex-specific cut-off points of each index for T2DM. Multiple logistic regression analysis was conducted and adjusted for age, BMI, hypertensive status, family history of diabetes, current smoking and alcohol consumption status, and regular exercise. The basic model was derived from the published diabetes risk model for middle-aged Koreans and included the variables listed above [10, 11]. We tested multicollinearity for all covariates based on the variable inflation factor (VIF). The area under the receiver operating characteristic curve (AROC) was calculated for each risk model of incident T2DM, indicating the diagnostic power of each model for incident T2DM during the follow-up period [28]. Differences in AROC between the basic model and each composite model were analyzed using the method of DeLong et al. [29]. Pencina et al. have suggested category-based net reclassification improvement (NRI) and integrated discrimination improvement (IDI) for calculating the usefulness of a new marker in prediction models [30]. The category-based NRI measures the accuracy of reclassification based on how well the subjects are reclassified as upwards for events and downwards for non-events. However, the category-based NRI can be affected by the number and choice of categories [31]. The continuous (category-free) NRI (cNRI) is an expanded method to solve limitation of the categories. They also proposed the IDI that calculates the extent of average sensitivity and ‘1-specificity’ when a new marker is added to the basic model [30]. We calculated the cNRI and IDI to compare the prediction and reclassification abilities of each measure when added to the basic model of diabetes. Macros were used to calculate cNRI and IDI, and data analysis was performed using SAS 9.4 and MedCalc [32].

## Results

### Baseline characteristics

Table 1 indicates the baseline characteristics of the study subjects. The ages of the subjects were 51.4 ± 8.6 years for men and 52.0 ± 8.9 years for women. The prevalence of hypertension was higher in men than in women (29.5% vs. 27.0%, *P* = 0.0149). By contrast, the percentage of subjects with a family history of diabetes was higher in women than in men (11.0% vs. 9.2%, *P* = 0.0067). There were also significant differences in anthropometric indices between sexes. Mean BMI and WC were 24.1 ± 2.9 kg/m^2^ and 83.3 ± 7.6 cm in men, and 24.7 ± 3.2 kg/m^2^ and 81.2 ± 9.5 cm in women, respectively. Mean TG, FPG, LAP, and TyG index were 170.8 ± 111.6 mg/dl, 84.5 ± 9.0 mg/dl, 38.1 ± 34.0, and 8.7 ± 0.5 in men, and 140.9 ± 75.6 mg/dl, 81.1 ± 7.7 mg/dl, 39.0 ± 30.6, and 8.5 ± 0.5 in women, respectively. TG-related indices also showed significant differences between the sexes, with the exception of LAP.

**Table 1:** Characteristics of the study population at baseline

Variables	Men	Women	*P-value*
	(*n* = 3636)	(*n* = 4072)	
Age, years	51.4 ± 8.6	52.0 ± 8.9	0.0011
Current Smoker, n(%)	1753 (48.2)	142 (3.5)	<.0001
Current Drinker, n(%)	2580 (71.0)	1085 (26.7)	<.0001
Hypertension, n(%)	1074 (29.5)	1101 (27.0)	0.0149
Family history of diabetes, n(%)	333 (9.2)	449 (11.0)	0.0067
Regular exercise, n(%)	1712 (47.1)	1654 (40.6)	<.0001
BMI, kg/m^2^	24.1 ± 2.9	24.7 ± 3.2	<.0001
WC, cm	83.3 ± 7.6	81.2 ± 9.5	<.0001
TG, mg/dl	170.8 ± 111.6	140.9 ± 75.6	<.0001
TyG index	8.7 ± 0.5	8.5 ± 0.5	<.0001
LAP	38.1 ± 34.0	39.0 ± 30.6	0.1925
SBP, mmHg	121.2 ± 16.6	119.9 ± 19.1	0.0029
DBP, mmHg	81.6 ± 10.8	78.5 ± 11.6	<.0001
FPG, mg/dl	84.5 ± 9.0	81.1 ± 7.7	<.0001
2hPG, mg/dl	110.5 ± 32.0	117.2 ± 28.3	<.0001
Total cholesterol, mg/dl	190.3 ± 34.6	188.9 ± 33.9	0.0670
HDL-C, mg/dl	43.8 ± 10.0	46.0 ± 10.0	<.0001
LDL-C, mg/dl	112.4 ± 34.2	114.7 ± 30.7	0.0017
HbA_1c_, mg/dl	5.6 ± 0.3	5.5 ± 0.4	0.1636

*P* values are from *t*-tests or chi-square tests for analysis of variance for continuous variables and categorical variables

Abbreviations: *BMI* body mass index, *WC* waist circumference, *TG* triglycerides; TyG index, the product of fasting glucose and triglycerides; *LAP* lipid accumulation product, *SBP* systolic blood pressure, *DBP* diastolic blood pressure, *FPG* fasting plasma glucose, *HDL-C* high-density lipoprotein cholesterol, *LDL-C* low density lipoprotein cholesterol, *HbA*_*1c*_ glycated hemoglobin

### Incidence of T2DM according to each index category

During the 10 year follow-up, the overall cumulative incidence of T2DM was 21.1% (766 cases) in men and 19.6% (797 cases) in women. Table 2 shows the overall cumulative incidence of T2DM, categorized by quartiles for each index. In men, the cumulative incidences of T2DM across the quartiles of TyG index were (lowest-highest) 13.3 and 31.5% and those of FPG were 12.9 and 35.9% and those of WC were 16.4 and 27.7%. In women, the values for TyG index were 11.1 and 30.9% and those FPG were 13.9 and 28.4% and those WC were 13.4 and 24.5%. The increase in the cumulative incidence of T2DM with higher category of WC was less marked than that with increasing TyG index or FPG.

**Table 2:** Number of incident type 2 diabetes cases according to the quartiles for each measure

Categories	Men (n = 3636)		Women (n = 4072)	
	Quartile	Diabetes (%)	Quartile	Diabetes (%)
WC (cm)	Q1 (< 78.0)	143 (16.4)	Q1 (< 74.0)	128 (13.4)
	Q2 (78.0–83.2)	162 (17.6)	Q2 (74.0–80.6)	198 (18.1)
	Q3 (83.3–88.2)	207 (22.3)	Q3 (80.7–87.8)	222 (22.2)
	Q4 (≥88.3)	254 (27.7)	Q4 (≥87.9)	249 (24.5)
TG (mg/dl)	Q1 (< 106.0)	137 (15.2)	Q1 (< 92.5)	126 (12.4)
	Q2 (106.0–142.9)	167 (18.4)	Q2 (92.5–121.9)	136 (13.6)
	Q3 (143.0–200.9)	196 (21.5)	Q3 (122.0–165.9)	225 (21.9)
	Q4 (≥201.0)	266 (29.0)	Q4 (≥166.0)	310 (30.3)
FPG (mg/dl)	Q1 (< 78.0)	106 (12.9)	Q1 (< 76.0)	133 (13.9)
	Q2 (78.0–82.9)	132 (14.7)	Q2 (76.0–79.9)	143 (16.3)
	Q3 (83.0–89.9)	190 (19.5)	Q3 (80.0–84.9)	206 (18.3)
	Q4 (≥90.0)	338 (35.9)	Q4 (≥85.0)	315 (28.4)
LAP	Q1 (< 16.7)	137 (15.1)	Q1 (< 18.8)	126 (12.4)
	Q2 (16.7–29.4)	140 (15.4)	Q2 (18.8–30.7)	167 (16.4)
	Q3 (29.5–49.1)	212 (23.3)	Q3 (30.8–50.1)	190 (18.7)
	Q4 (≥49.2)	277 (30.5)	Q4 (≥50.2)	314 (30.8)
TyG index	Q1 (< 8.4)	121 (13.3)	Q1 (< 8.2)	113 (11.1)
	Q2 (8.4–8.6)	153 (16.8)	Q2 (8.2–8.4)	140 (13.8)
	Q3 (8.7–9.0)	206 (22.7)	Q3 (8.5–8.7)	229 (22.5)
	Q4 (≥9.1)	286 (31.5)	Q4 (≥8.8)	315 (30.9)

Abbreviations: *WC* waist circumference, *TG* triglycerides, *FPG* fasting plasma glucose, *LAP* lipid accumulation product, *TyG index* the product of fasting glucose and triglycerides

### Cut-off points of each index for predicting T2DM

Table 3 shows the AROC values and cut-off points of the indices for predicting T2DM. The AROCs for WC, TG, FPG, LAP, and TyG index were 0.579, 0.592, 0.660, 0.602, and 0.623 in men 0.576, 0.627, 0.599, 0.623, and 0.644 in women respectively. The cut-off points for predicting T2DM were 84.00 cm, 172.00 mg/dl, 87.00 mg/dl, 30.50, and 8.86 in men and 78.17 cm, 122.00 mg/dl, 84.00 mg/dl, 35.84, and 8.52 in women for WC, TG, FPG, LAP, and TyG index, respectively.

**Table 3:** The area under the ROC curve (AROC) and cut-off points for indices to predict type 2 diabetes

Index	AROC (95% CI)	Cut-off point	Sensitivity (%)	Specificity (%)	Youden index
Men					
WC (cm)	0.579 (0.563–0.595)	84.00	56.01	57.11	0.13
TG (mg/dl)	0.592 (0.576–0.608)	172.00	46.74	67.84	0.15
FPG (mg/dl)	0.660 (0.645–0.676)	87.00	51.31	72.89	0.24
LAP	0.602 (0.586–0.618)	30.50	62.79	55.54	0.18
TyG index	0.623 (0.607–0.638)	8.86	52.09	66.59	0.19
Women					
WC (cm)	0.576 (0.561–0.592)	78.17	69.26	43.54	0.13
TG (mg/dl)	0.627 (0.612–0.642)	122.00	66.37	54.41	0.21
FPG (mg/dl)	0.599 (0.584–0.614)	84.00	39.52	75.69	0.15
LAP	0.623 (0.607–0.637)	35.84	57.59	61.59	0.19
TyG index	0.644 (0.629–0.659)	8.52	67.25	55.85	0.23

Abbreviations: *AROC* area under the receiver operating characteristic curve, *WC* waist circumference, *TG* triglycerides, *FPG* fasting plasma glucose, *LAP* lipid accumulation product, TyG index, the product of fasting glucose and triglycerides

### Odds ratios for incident T2DM for each composite predictive model

Table 4 shows the odds ratio (OR) for incident T2DM in higher quartiles compared to the first quartile of each index. The unadjusted ORs for all TG-related indices for incident T2DM were higher than that of WC. These trends were similar after adjustment for age, BMI, hypertensive status, family history of diabetes, smoking, alcohol consumption status and regular exercise. When the highest quartile for each index was compared to the lowest, the adjusted ORs of WC, TG, FPG, LAP, and TyG index were 1.64 (95% confidence interval (CI), 1.13–2.38), 2.03 (1.59–2.61), 3.85 (2.99–4.97), 2.47 (1.82–3.34), and 2.79 (2.16–3.60) in men, and 1.17 (0.83–1.65), 2.42 (1.90–3.08), 2.15 (1.71–2.71), 2.44 (1.82–3.26), and 2.85 (2.22–3.66) in women, respectively. The calculated VIF for all covariates in the multivariate model, were below 5.0, indicating no severe multicollinearity among covariates [33].

**Table 4:** Multiple logistic regression analyses for each measure in predicting type 2 diabetes (odds ratios and 95% confidence intervals)

Categories	Unadjusted					Adjusted^a)^				
	WC	TG	FPG	LAP	TyG index	WC	TG	FPG	LAP	TyG index
	ORs (95% CI)	ORs (95% CI)	ORs (95% CI)	ORs (95% CI)	ORs (95% CI)	ORs (95% CI)	ORs (95% CI)	ORs (95% CI)	ORs (95% CI)	ORs (95% CI)
Men										
Q1	Ref.	Ref.	Ref.	Ref.	Ref.	Ref.	Ref.	Ref.	Ref.	Ref.
Q2	1.09 (0.85–1.39)	1.26 (0.99–1.62)	1.16 (0.88–1.53)	1.03 (0.79–1.33)	1.32 (1.02–1.70)	1.07 (0.81–1.40)	1.21 (0.94–1.55)	1.18 (0.89–1.56)	1.04 (0.79–1.36)	1.26 (0.97–1.64)
Q3	1.46 (1.15–1.85)	1.54 (1.21–1.95)	1.64 (1.27–2.12)	1.71 (1.35–2.18)	1.91 (1.49–2.45)	1.35 (1.00–1.83)	1.39 (1.08–1.79)	1.68 (1.29–2.19)	1.70 (1.28–2.25)	1.82 (1.41–2.36)
Q4	1.95 (1.55–2.46)	2.29 (1.82–2.88)	3.78 (2.96–4.82)	2.47 (1.96–3.11)	2.99 (2.36–3.79)	1.64 (1.13–2.38)	2.03 (1.59–2.61)	3.85 (2.99–4.97)	2.47 (1.82–3.34)	2.79 (2.16–3.60)
Women										
Q1	Ref.	Ref.	Ref.	Ref.	Ref.	Ref.	Ref.	Ref.	Ref.	Ref.
Q2	1.43 (1.12–1.82)	1.11 (0.86–1.44)	1.20 (0.93–1.55)	1.39 (1.08–1.78)	1.28 (0.98–1.66)	1.14 (0.88–1.48)	1.02 (0.78–1.33)	1.18 (0.91–1.53)	1.26 (0.97–1.64)	1.19 (0.91–1.55)
Q3	1.86 (1.46–2.36)	1.98 (1.56–2.51)	1.38 (1.09–1.75)	1.63 (1.27–2.07)	2.32 (1.82–2.97)	1.27 (0.95–1.69)	1.69 (1.32–2.16)	1.32 (1.04–1.68)	1.35 (1.03–1.78)	1.97 (1.53–2.53)
Q4	2.10 (1.66–2.66)	3.07 (2.44–3.87)	2.45 (1.95–3.06)	3.15 (2.51–3.97)	3.59 (2.83–4.55)	1.17 (0.83–1.65)	2.42 (1.90–3.08)	2.15 (1.71–2.71)	2.44 (1.82–3.26)	2.85 (2.22–3.66)

^a)^Adjusted for age, body mass index, status of hypertension, family history of diabetes, smoking status, alcohol consumption, and regular exercise

Abbreviations: *ORs* odds ratios, *WC* waist circumference, *TG* triglycerides, *FPG* fasting plasma glucose, *LAP* lipid accumulation product, *TyG index*, the product of fasting glucose and triglycerides

### Effects of the addition of each index to the basic model of T2DM on cNRI, IDI, and AROC

Table 5 shows the reclassification and discrimination abilities when each measure was added to the basic model of diabetes. The AROCs for the addition of TyG index or FPG to the basic model were larger than those for the other indices in both sexes. The cNRI for TG, FPG, LAP, and TyG index were 22.7% (*P* < 0.0001), 48.0% (*P* < 0.0001), 23.6% (*P* < 0.0001), and 38.7% (*P* < 0.0001) in men, and 28.6% (*P* < 0.0001), 21.3% (*P* < 0.0001), 21.3% (*P* < 0.0001), and 36.0% (*P* < 0.0001) in women, respectively. By contrast, the addition of WC made cNRI only 5.6% (*P* = 0.1689), in men and − 2.3% (*P* = 0.5666), in women. The IDI was also higher when TG-related indices or FPG was added than when WC was added to the basic model of T2DM in both sexes.

**Table 5:** Reclassification and discrimination results associated with the risk prediction of incident type 2 diabetes according to each measure

Parameter	Men						Women					
	AROC	*p*-value*	cNRI	*p*-value	IDI	*p*-value	AROC	*p*-value*	cNRI	*p*-value	IDI	*p*-value
Basic model^a)^	0.615						0.621					
Basic model + WC	0.619	0.1176	0.056	0.1689	0.002	0.0140	0.621	0.3233	−0.023	0.5666	0.000	1.0000
Basic model + TG	0.632	<.0001	0.227	<.0001	0.007	<.0001	0.652	<.0001	0.286	<.0001	0.019	<.0001
Basic model + FPG	0.692	<.0001	0.480	<.0001	0.064	<.0001	0.652	<.0001	0.214	<.0001	0.018	<.0001
Basic model + LAP	0.634	<.0001	0.236	<.0001	0.007	<.0001	0.644	<.0001	0.213	<.0001	0.016	<.0001
Basic model + TyG index	0.656	<.0001	0.387	<.0001	0.023	<.0001	0.666	<.0001	0.360	<.0001	0.029	<.0001

^a)^Basic model: age, body mass index, status of hypertension, family history of diabetes, smoking status, alcohol consumption, and regular exercise

*P-value for AROC means vs. Basic model

Abbreviations: *AROC* area under the receiver operating characteristic curve, *WC* waist circumference, *TG* triglycerides, *LAP* lipid accumulation product, *TyG index* the product of fasting glucose and triglycerides, *cNRI* continuous net reclassification improvement, *IDI* Integrated Discrimination Improvement

## Discussion

We showed that the predictive ability of the simple T2DM risk model was increased when TG-related indices or FPG was added. Recently, several diabetes risk models have been proposed to screen high-risk groups, some of which included blood parameters. WC was included as one of the components in the model because increased WC increases T2DM risk [8]. However, WC is not easily used in practice because of measurement inaccuracy [34]. Among the TG-related indices analyzed in the present study, the AROC and reclassification ability of TyG index were higher than for TG or LAP. Although the inclusion of LAP also improved the predictive ability of the risk model for incident diabetes, this required the additional inclusion of WC, while this was not required to demonstrate improvements in the risk model by the inclusion of TG or TyG index [20]. Most studies that used TyG index to predict incident T2DM used it as a surrogate for insulin resistance, and reported that its predictive ability is better than that of the homeostasis model of assessment (HOMA-IR) [19, 35, 36]. However, Abbasi and Reaven showed that the correlation between insulin-mediated glucose uptake (IMGU) and TyG index is not better than that between IMGU and TG or HOMA-IR [37, 38]. Nevertheless, it was noted that TyG index is a practical measure for use in T2DM prediction because of its cost-efficiency [37]. Moreover, the use of HbA_1c_ has been recommended for diagnosis and screening of patients. Lim et al. added HbA_1c_ to a diabetes risk model and demonstrated an increase in predictive ability for T2DM in a middle-aged Korean cohort, while Ahn et al. also demonstrated through longitudinal validation analysis that adding FPG or HbA_1c_ to the simple diabetes risk model also improves its predictive ability [11, 22]. Thus, in general, the predictive ability of a diabetes risk model that includes laboratory parameters such as TG, FPG, or HbA_1c_ is better than that of the simple diabetes risk model [11, 14, 22].

The advantages of the simple diabetes risk model are that it is inexpensive and patients can use it for self-assessment [8]. Weighed against the improved predictive accuracy obtained by the addition of HbA_1c_ and the Oral Glucose Tolerance Test (OGTT) to the T2DM risk model, there are substantial practical constraints on screening the whole population using such measurements [22]. As an effective alternative, a two-pronged approach to screening has been proposed [22, 39]. This approach consists of using the non-laboratory score for the general population and using the laboratory score for patients in a higher-risk group for T2DM. For screening as part of the Korean NHI program, TG-related indices or FPG are more suitable as one of the components of the T2DM risk model than OGTT or HbA_1c_, given that these measurements are not currently included in primary screening measurements in Korea [23]. To the best of our knowledge, there is no TyG index criterion yet. On the other hand, several studies conducted in Europe and Asian have shown that the risk of incident T2DM was increased with increasing TyG index [40–42]. Some researchers have proposed a cut-off value of TyG index of 8.8 for incident T2DM and insulin resistance [41, 42]. The subjects in the present study were classified by the cut-off value of TyG index of 8.8. The adjusted odds ratio (95% CI) of incident T2DM in the subjects with TyG index ≥8.8 was 1.95 (1.73–2.20) compared to the counterparts. We also have proposed sex-specific cut-off points of TyG index (≥ 8.86 in men, ≥ 8.52 in women) as predictors of T2DM based on the maximized Youden index. Compared to the counterparts, the adjusted odds ratio (95% CI) of incident T2DM in the men with TyG index ≥8.86 and in the women with TyG index ≥8.52 were 2.01 (1.69–2.39) and 2.16 (1.82–2.56), respectively (Appendix 1). Considering NIH’s universe coverage, the health promotion that offered the general health check-ups for 17.6 million Koreans in 2016, and its following examination rate (77.7%), there should be less burden caused by blood tests than other countries [43]. Therefore, TyG index is recommended as a screening tool for the prediction of T2DM. Although TyG index predicts incident T2DM well, its usefulness is inconsistent, when compared to fasting glucose [38, 44]. For predicting T2DM risk, TyG index was not better than FPG or OGTT in Isfahan Diabetes Prevention Study [38]. Wang et al. reported that TyG index, LAP, and visceral adiposity were not superior to FPG or WC alone as diabetes predictors among Chinese [44]. In the Vascular-Metabolic CUN cohort, Navarro-Gonzalez et al. compared the prediction ability of TyG index and FPG for onset T2DM [35]. Association between indexes and their discrimination for onset T2DM were different depending on the fasting glucose subgroup. When the highest quartile for each index was compared to the lowest, the hazard ratios (HRs) of TyG index and FPG were 3.0 and 7.3 in the impaired fasting glucose group. On the other hand, TyG index showed stronger association with onset DM than FPG in the normal fasting glucose group (HRs: 6.8 vs. 4.6). The discrimination of TyG index for onset T2DM was also better than that of FPG in the normal fasting glucose group (AROC: 0.75 vs. 0.66). In the present study, we found the association between some metabolic syndrome (MetS) components and incident T2DM (Appendix 2). Therefore, we compared the discrimination of each index for incident T2DM in the stratified MetS components (Appendix 3). In the elevated FPG group, the discrimination of FPG (AROC: 0.506) for incident T2DM was inferior to that of other indices. On the other hand, TyG index was a more robust discrimination index than other indices in the group stratified by sex and MetS components. Our findings indicate that TyG index is not only a better predictor for incident diabetes than WC, LAP, and TG, but it also has a better reclassification ability. On the other hand, association between FPG and its(their) reclassification ability for incident T2DM were different depending on sex. The present study used community-based long-term prospective cohort data. Therefore, the temporal relationship between each measure and incident diabetes is clear. Our findings indicate that TyG index is not only a better predictor for incident diabetes than WC, LAP, and TG, but it also has a better reclassification ability. It was also noted that association between FPG and its reclassification ability for incident T2DM were different depending on sex. There were, however, a few limitations to this study. Firstly, even though KoGES is designed to include subjects who live both in rural and urban areas, the data are not representative of the entire Korean population [45]. Secondly, dietary intake was not included in the analysis. Moreover, Baik et al. had reported that the usefulness of dietary information in cardiovascular disease risk prediction models [46]. However, dietary information has been excluded from the questionnaire in medical check since 2009. Finally, our results were not validated using a separate Korean dataset. Therefore, additional studies are required to validate our data.

## Conclusions

In conclusion, TG-related indices and FPG were more accurate than WC in the prediction of incident T2DM. In the subgroup categorized by sex and fasting glucose level, TyG index was a more robust predictor for onset T2DM than other indexes. Therefore, TyG index can be a useful screening tool for incident T2DM in middle-aged Koreans.

## Appendix 1

**Table 6:** Multiple logistic regression analyses for cutoff-points of TyG index in predicting type 2 diabetes (odds ratios and 95% confidence intervals)

Cutoff-points	Men		Women	
	Unadjusted	Adjusted^a)^	Unadjusted	Adjusted^a)^
	ORs (95% CI)	ORs (95% CI)	ORs (95% CI)	ORs (95% CI)
TyG index ≥8.8^b)^	2.05 (1.75–2.41)	1.93 (1.62–2.29)	2.41 (2.05–2.83)	2.03 (1.71–2.41)
TyG index ≥8.86/8.52^c)^	2.13 (1.82–2.51)	2.01 (1.69–2.39)	2.57 (2.18–3.02)	2.16 (1.82–2.56)

^a)^Adjusted for age, body mass index, status of hypertension, family history of diabetes, smoking status, alcohol consumption, and regular exercise, ^b)^TyG index ≥8.8 cm, Lee et al. [42], ^c)^TyG index ≥8.86 cm for men ≥8.52 for women in the present study

Abbreviations: *ORs* odds ratios; TyG index, the product of fasting glucose and triglycerides

## Appendix 2

**Table 7:** Multiple logistic regression analyses for each MetS component in predicting type 2 diabetes (odds ratios and 95% confidence intervals)

Components of MetS^a)^	Men		Women	
	Unadjusted	Adjusted ^h)^	Unadjusted	Adjusted ^h)^
	ORs (95% CI)	ORs (95% CI)	ORs (95% CI)	ORs (95% CI)
Central obesity^b)^	1.71 (1.42–2.06)	1.35 (1.10–1.65)	1.56 (1.33–1.83)	1.16 (0.97–1.38)
Elevated TG^c)^	1.70 (1.45–2.00)	1.57 (1.31–1.87)	2.26 (1.93–2.64)	1.90 (1.60–2.26)
Low HDL-C^d)^	1.14 (0.96–1.34)	1.01 (0.84–1.22)	1.53 (1.28–1.83)	1.21 (1.00–1.47)
Elevated FPG^e)^	5.43 (4.09–7.21)	5.16 (3.85–6.92)	3.65 (2.39–5.57)	3.55 (2.29–5.50)
Elevated BP^f)^	1.79 (1.52–2.10)	1.47 (1.24–1.75)	1.65 (1.41–1.93)	1.29 (1.09–1.54)
MetS^g)^	2.22 (1.86–2.66)	2.24 (1.87–2.69)^i)^	2.22 (1.88–2.61)	2.03 (1.70–2.41)^i)^

^a)^Modified NCEP-ATP III criteria [47] with the Korean cut-off for WC, ^b)^WC ≥ 90 cm for men ≥85 for women, Lee et al. [48], ^c)^TG ≥ 150 mg/dl, ^d)^HDL-C < 40 mg/dl for men < 50 mg/dl for women, ^e)^FPG ≥ 100 mg/dl, ^f)^Systolic blood pressure ≥ 130 mmHg or diastolic blood pressure ≥ 85 mmHg, ^g)^Number of MetS components ≥3, ^h)^Adjusted for other components of MetS, age, family history of diabetes, smoking status, alcohol consumption, and regular exercise, ^i)^Adjusted for age, family history of diabetes, smoking status, alcohol consumption, and regular exercise

Abbreviations: *MetS* Metabolic syndrome, *ORs* odds ratios, *TG* triglyceride, *HDL-C* high-density lipoprotein cholesterol, *FPG* fasting plasma glucose, *BP* blood pressure, *WC*, waist circumference

## Appendix 3

**Table 8:** The area under the ROC curve (AROC) for indices to predict type 2 diabetes stratified by MetS components

MetS components^a)^	Central obesity^b)^		Elevated TG^c)^		Low HDL-C^d)^		Elevated FPG^e)^		Elevated BP^f)^	
Subgroup	No	Yes	No	Yes	No	Yes	No	Yes	No	Yes
Index	AROC	AROC	AROC	AROC	AROC	AROC	AROC	AROC	AROC	AROC
Men	(*n* = 2929)	(*n* = 707)	(*n* = 1970)	(*n* = 1666)	(*n* = 2326)	(*n* = 1310)	(*n* = 3423)	(*n* = 213)	(*n* = 2092)	(*n* = 1544)
WC	0.553	0.532	0.535	0.569	0.570	0.584	0.571	0.573	0.565	0.559
TG	0.589	0.534	0.546	0.559	0.598	0.574	0.588	0.591	0.572	0.588
FPG	0.648	0.680	0.637	0.676	0.661	0.666	0.621	0.506	0.649	0.661
LAP	0.584	0.537	0.546	0.582	0.598	0.599	0.595	0.604	0.582	0.591
TyG index	0.618	0.569	0.605	0.607	0.631	0.605	0.607	0.586	0.602	0.620
Women	(*n* = 2680)	(*n* = 1392)	(*n* = 2754)	(*n* = 1318)	(*n* = 1276)	(*n* = 2796)	(*n* = 3983)	(*n* = 89)	(*n* = 2639)	(*n* = 1433)
WC	0.556	0.519	0.536	0.564	0.520	0.583	0.576	0.506	0.570	0.545
TG	0.610	0.626	0.567	0.559	0.611	0.622	0.629	0.596	0.614	0.621
FPG	0.590	0.598	0.581	0.617	0.550	0.616	0.586	0.566	0.598	0.587
LAP	0.601	0.625	0.553	0.590	0.565	0.630	0.623	0.570	0.608	0.609
TyG index	0.628	0.643	0.595	0.596	0.621	0.643	0.641	0.597	0.633	0.634

^a)^Modified NCEP-ATP III criteria [47] with the Korean cut-off for WC, ^b)^WC ≥ 90 cm for men ≥85 for women, Lee et al. [48], ^c)^TG ≥ 150 mg/dl, ^d)^HDL-C < 40 mg/dl for men < 50 mg/dl for women, ^e)^FPG ≥ 100 mg/dl, ^f)^ Systolic blood pressure ≥ 130 mmHg or diastolic blood pressure ≥ 85 mmHg

Abbreviations: *MetS* Metabolic syndrome, *TG* triglyceride, *HDL-C* high-density lipoprotein cholesterol, *FPG* fasting plasma glucose, *BP* blood pressure, *AROC* area under the receiver operating characteristic curve, *WC* waist circumference, *LAP* lipid accumulation product, *TyG index*, the product of fasting glucose and triglycerides

## Abbreviations

AROC: Area under the receiver operating characteristic curve
BMI: Body mass index
CI: Confidence interval
cNRI: Continuous net reclassification improvement
FPG: Fasting plasma glucose
HbA_1c_: Glycated hemoglobin
HRs: Hazard ratios
IDI: Integrated discrimination improvement
IMGU: Insulin-mediated glucose uptake
KNHANES: Korea National Health and Nutrition Examination
KNIH: Korea National Institute of Health
KoGES: Korean Genome and Epidemiology Study
LAP: Lipid accumulation product
NHI: National Health Insurance
OGTT: Oral Glucose Tolerance Test
ORs: Odds ratios
T2DM: Type 2 diabetes mellitus
TG: Triglyceride
TyG index: Product of fasting glucose and triglyceride
VIF: Variable inflation factor
WC: Waist circumference
